# Selective ablation of cochlear hair cells promotes engraftment of human embryonic stem cell-derived progenitors in the mouse organ of Corti

**DOI:** 10.1186/s13287-021-02403-9

**Published:** 2021-06-19

**Authors:** Hiroki Takeda, Anna Dondzillo, Jessica A. Randall, Samuel P. Gubbels

**Affiliations:** 1 Department of Otolaryngology, University of Colorado Denver, Academic Office One, Suite 3001, 12631 E 17th Avenue, MS B205, Aurora, CO 80045 USA; 2 Department of Otolaryngology-Head and Neck Surgery, Kumamoto University, Graduate School of Medicine, Kumamoto City, Japan

**Keywords:** ES cell, Cell transplantation, Selective ablation, Regeneration, Cochlea

## Abstract

**Background:**

Hearing loss affects 25% of the population at ages 60–69 years. Loss of the hair cells of the inner ear commonly underlies deafness and once lost this cell type cannot spontaneously regenerate in higher vertebrates. As a result, there is a need for the development of regenerative strategies to replace hair cells once lost. Stem cell-based therapies are one such strategy and offer promise for cell replacement in a variety of tissues. A number of investigators have previously demonstrated successful implantation, and certain level of regeneration of hair and supporting cells in both avian and mammalian models using rodent pluripotent stem cells. However, the ability of human stem cells to engraft and generate differentiated cell types in the inner ear is not well understood.

**Methods:**

We differentiate human pluripotent stem cells to the pre-placodal stage in vitro then transplant them into the mouse cochlea after selective and complete lesioning of the endogenous population of hair cells.

**Results:**

We demonstrate that hair cell ablation prior to transplantation leads to increased engraftment in the auditory sensory epithelium, the organ of Corti, as well as differentiation of transplanted cells into hair and supporting cell immunophenotypes.

**Conclusion:**

We have demonstrated the feasibility of human stem cell engraftment into an ablated mouse organ of Corti.

**Graphical abstract:**

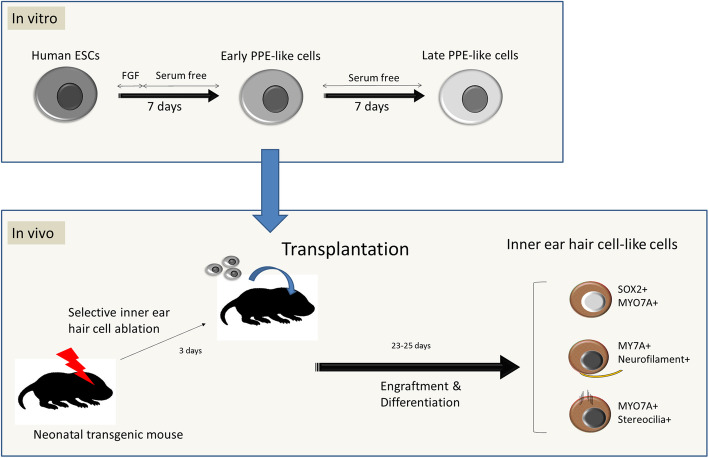

**Supplementary Information:**

The online version contains supplementary material available at 10.1186/s13287-021-02403-9.

## Background

Hearing loss is most commonly caused by inner ear cochlear hair cell (HC) death, and the resulting hearing deficits have a profound, negative impact on the daily life of affected people. Furthermore, mammalian hearing loss is irreversible, because mammalian HCs, unlike those of birds [1–3], do not regenerate spontaneously. Therefore, the development of the methods for hair cell regeneration or replacement would help in establishing curative therapies for sensory hearing loss in the future. The use of pluripotent stem cells for this purpose represents one potential promising such method. One strategy for stem cell-based therapies involves in vitro generation of the inner ear (otic) progenitor cells followed by their subsequent transplantation into the deafened cochlea (in-depth review [4]). Successful generation of otic progenitor and inner ear hair cells from mouse [5] or human stem cells [6–8] has been elegantly demonstrated in vitro. Furthermore, human neuronal progenitor cells have been successfully engrafted into the injured gerbil modiolus leading to restoration of hearing [6]. Other xenograft models combining an avian recipient of mammalian stem cells showed successful engraftment and differentiation of mouse stem cell-derived progenitor cells into the nascent chick auditory sensory epithelium [9, 10]. However to date, little is known of the ability of human cells to engraft in the mammalian auditory sensory epithelium, the organ of Corti (OC) [11, 12]. Multiple investigators have reported successful engraftment of mouse stem cells in the mouse [13], guinea pig [14], and rat OC [15, 16], with the consistent finding of only a small number of cells engrafting successfully. Here we test the plausibility of grafting human pluripotent stem cell-derived otic progenitors into a murine cochlea after induced hair cell loss. We focus our investigations on transplanting stem cells at an early otic fate, and therefore, we differentiated human embryonic stem cells (hESC) to pre-placodal ectoderm (PPE) using previously reported methods [17].

We used a round window (RW) injection into the scala tympani perilymph as our route of delivery of the cells to increase the chances of cell survival (avoiding high K+ environment of the scala media endolymph) and migration into the OC through the basilar membrane. Based on the existing literature on the successful engraftment at an early age in the chick inner ear [9, 10], we used neonatal mice as transplantation recipients. Furthermore, as Lang and colleagues have demonstrated that the ablated sensory epithelium may be more amenable to the migration and engraftment of transplanted cells [18], we sought to use an acutely deafened model in our studies. To do this, we used Pou4f3DTR/+, an established mouse model for acute cochlear hair cell loss [19] to induce hair cell loss with diphtheria toxin injections. Using this model as well as wild type (WT) controls, we assess the survival and engraftment of transplanted human pluripotent stem cell-derived otic progenitors.

The goal of these experiments was to establish the feasibility of human stem cell engraftment in the mouse cochlea in vivo. Our hypothesis is that acute lesioning of the murine OC will allow for improved survival, engraftment, and terminal differentiation of transplanted human pluripotent stem cell-derived otic progenitors.

## Methods

### Animal model and deafening procedure

Animal work was approved by the Institutional Animal Care and Use Committee of the University of Colorado Anschutz Medical Campus.

Pou4f3^tm1.1(HBEGF)Jsto^ mice on a C57BL/6 J background (from now on, we refer to them as Pou4f3DTR/+ mice) were a kind gift from Dr. Rubel’s lab [20]. Additional Pou4f3^tm1.1(HBEGF)Jsto^ mice were purchased from the Jackson Laboratory (stock no: 028673). The coding region of human diphtheria toxin receptor (HBEGF) was introduced upstream of the translation initiation site of the mouse Pou4f3 (POU domain, class 4, transcription factor 3) gene. The human diphtheria toxin receptor increases the sensitivity of mouse cells to the toxin. Given that the Pou4f3 genes are only expressed in the inner ear hair cells, DT injected into a mouse specifically ablates inner ear hair cells and spares all other cells in the organ of Corti and beyond. The mouse colony was maintained by cross-breeding: Pou4f3DTR/+ and Pou4f3DTR/+, or Pou4f3DTR/+ and wild type (WT) mice. Mice at postnatal day (P) 1–2 were genotyped. The following primers were used for genotyping: wild type reverse [5′-ATT GTT CTG GGC GAC ATG A-3′], common [5′-AAG AAG CAG GTG GGG GAG AG-3′] and mutant reverse [5′-CAG AAA GAG CTT CAG CAC CAC-3′]. In order to ablate hair cells, 2–4 ng/g DT (2 ng/g BW for P1 and 4 ng/g BW for P2 mice) was intramuscularly injected into the right caudal thigh muscles for both WT and Pou4f3DTR/+ mice at P1–2 after genotyping.

There were a total of 40 animals (*n* = 40) used in this study. Twenty animals were used to establish diphtheria toxin effects on the cochleae of wild type non-treated (*n* = 4), wild type treated (*n* = 5), Pou4f3DTR/+ non-treated (*n* = 5), and Pou4f3DTR/+ treated (*n* = 6) animals (Fig. 2). Ten wild type animals were used to study stem cell transplantation and survival after 7 days (*n* = 6) and 14 days (*n* = 4) post hESCs delivery (Fig. 3). Finally, ten Pou4f3DTR/+ animals were used to test the efficacy of stem cell engraftment and survival in the cochleae with ablated hair cells. This experiment consisted of three different survival time groups: 7 days (*n* = 4), 14 days (*n* = 3), and 26–28 days (*n* = 3) of incubation time after stem cell delivery (Figs. 4 and 5).

### Cell culture of human ES cells

We used two cell lines: WA09 (H9 cell line from WiCell) [21] for in vitro cell differentiation study and LT2e-H9CAGGFP (GFP-expressing H9 cell line from WiCell) [22] for mainly in vivo cell transplantation study. In the LT2e-H9CAGGFP cell line, the green fluorescent protein (GFP) gene is driven by a CAG promoter, which results in constitutive and robust GFP expression for a prolonged time period. Both cell lines were maintained on Matrigel-coated plates in mTeSR1 medium (STEMCELL, Vancouver, Canada) until the start of the day of differentiation (day 0). The medium was changed daily except for one double feed during a 7-day period. Cells were passaged every 3 to 4 days.

For differentiation of the stem cells to the pre-placodal ectoderm (PPE) state, we used Leung et al.’s protocol [17]. For 48–72 h, cells were cultured on Matrigel-covered plates and maintained in Dulbecco’s modified Eagle’s medium: Nutrient Mixture F-12 (DMEM/F12) supplemented with 20% KnockOut serum replacement, 1 x Non-Essential Amino Acid (NEAA), 1 x L-Glutamine, 100uM Beta-mercaptoethanol, and 8 ng/ml basic Fibroblast Growth Factor (bFGF). Ten micromolar Rho kinase inhibitor (Y27632, Calbiochem, San Diego, CA) was included for the first 24 h and then removed. On day 3, the medium was changed to serum-free (SF) media containing 1% N2 supplement, 2% B27 supplement, 1 × NEAA, 1 × L-glutamine, and 100 μM β-mercaptoethanol in DMEM/F12, and the culture was maintained in this media until the day of harvest. This medium was changed daily for the first 6 days and every other day thereafter.

For transplantation, LT2e hESCs were differentiated for 7–8 days prior to transplantation with the same protocol as WA09 hESCs described above. On the day of transplantation, differentiated cells were dissociated into a single-cell suspension by incubating with Accutase (StemCell, Vancouver, Canada) for 6 min at room temperature and then triturated (cell suspension was pipetted up and down several times). Cells were centrifuged and adjusted to the concentration of 5.0–10.0 × 10^6^/ml in SF media.

### Semi-quantitative PCR

mRNA was extracted from cells at each day of differentiation using a RNeasy Mini Kit® (QIAGEN, Germantown, MD). mRNA was then quantitated using Qubit and 1 μg of mRNA was converted to DNA using the qScript cDNA Synthesis Kit® (Quanta bio, Beverly, MA). Subsequently, DNA was PCR amplified (ProFlex PCR System; Thermo Fisher, Waltham, MA) with GoTaq® master mix (Promega, Madison, WI). The amplified DNA was then submitted to electrophoresis on 2% agarose gels and PCR bands were visualized with an ultraviolet transilluminator. Images were captured and stored on a computer. For quantification of gene expression levels, the detected bands were analyzed with Image J software (NIH, Maryland, MD), and we obtained relative intensity by dividing the intensity of each band by that of a 500-bp ladder band on the same gel in order to reduce bias in combining data from each sample taken with different UV light exposure time.

### Technical considerations of the transplant procedure

To assess cell viability for cell transplantation, we examined cell survival rate at the three technical stages: (1) after cell dissociation and collection, (2) in a suspension during surgery time until injection, and (3) after ejection from the glass pipettes (using several different tip sizes and the same pressure setting as our cell transplantation experiment). We found that when the pipette tip size was 12.5 μm, cells completely clogged the tip, and with a tip size of 18.75 μm, about half of the ejected cells died as assessed by cell viability test using Trypan blue (Additional Figure 4). Almost all cells survived, however, when ejected through the tip of 25 μm diameter (Additional Figure 4A). Thus we used glass pipettes with a 25-μm diameter tip opening for all the following transplantation studies. Furthermore, we found that SF media is comparable or perhaps better as a solution for a short-term cell storage during the surgery just before the injection (Additional Figure 4B). Moreover, we found there was no real difference in cell viability when incubating the cells at various temperatures (*n* = 1) (Additional Figure 4C). Therefore, we kept cells at room temperature from the time of cell dissociation and during the surgery until the time of transplantation.

### Cell transplantation

Differentiation of LT2e hESCs started 7–8 days prior to the day of transplantation to match the timing of cell differentiation and transplantation. Glass pipettes (Harvard Apparatus, Holliston, MA; 30-0057) were pulled with micropipette pullers P-1000 (Sutter Instrument, Novato, CA), and then the tips of the glass pipettes were beveled with a Microelectrode Beveler BV-10 (Sutter Instrument, Novato, CA) and adjusted to 25 μm tip opening before the day of transplantation. On the day of transplantation, neonatal mice at 3 days after DT injection were anesthetized on ice (hypothermia). After the induction of anesthesia and once the anesthetized had reached an adequate anesthetic depth, the skin on the left side of the post-auricular region was sterilized with betadine 1% iodine solution. The right ear served as an untreated control. Under a stereomicroscope (Leica M205A, Allendale, NJ), a 0.5 cm post-auricular incision was made behind the left ear, and then subcutaneous muscles were incised and separated to expose the temporal bone. The left otic bulla was opened using forceps to expose the round window niche, and then the hole was widened sufficiently to visualize the round window membrane (RWM). The cell suspension was injected from a glass pipette into the scala tympani through the round window membrane by a Picoliter microinjector PLI-100A (Warner Instrument, Hamden, CT) for 1 to 2 min. A total of approximately 0.5 μl (5.0–10.0 × 10^6^ cells/ml, 2.5–5.0 × 10^3^ cells/injection) was delivered to the cochlea. The punctured RWM was sealed quickly after pulling out the pipette with a small piece of muscle or adipose tissue harvested from the cervical area during the approach to prevent leakage from the round window at the puncture site. The hole in the auditory bulla was also covered with adipose tissue and the wound was sutured in layers with a 6-0 absorbable chromic suture. The animals were kept on a heated pad until they recovered and were placed back in the cage with their mothers.

### Immunocytochemistry and immunohistochemistry

For immunocytochemistry, cells at each day of differentiation were washed by 1xPBS and then fixed with 4% paraformaldehyde for 15 min and washed three times with PBS. After blocking (0.3% TritonX, 1% bovine serum albumin, and 10% normal donkey or goat serum) for 30 min, the slides were incubated with primary antibodies (Additional Table 1) overnight at 4 °C. The following day, slides were washed 3X with PBS and then incubated with secondary anti-rabbit/mouse/goat Alexa Fluor 488/546/647 (Invitrogen, Carlsbad, CA) antibodies for 1 h at room temperature in the dark. The slides were then washed 3 times with PBS and coverslipped with mounting medium with DAPI (Southern Biotech, Birmingham, AL or Vector, Burlingame, CA).

At each day of harvest, the treated animals were sacrificed with an overdose of the intraperitoneal administration of xylazine and ketamine-HCl mix followed by decapitation. Cochleae were extracted and fixed with 4% paraformaldehyde overnight at 4 °C. EDTA solution (0.12 M) was used for decalcification of the bone structure (P8-9: for 2–4 h, P15 to P16: overnight, P27 to P30: for 2–3 days). For frozen serial sections, the cochleae were mounted in optimal cutting temperature compound (OCT) and frozen at − 80 °C freezer then stored at − 20 °C. The cochleae were cut on a cryostat (LEICA CM1850, Allendale, NJ) into 8–10 μm slices that were collected onto the glass slides. The entire cochlea was sliced and sections collected on the Superfrost Excell microscope slides (Fisherbrand, Cat # 22-034-985). Mounted slides were washed with PBS, and to increase tissue adherence to the glass, a 4% paraformaldehyde was applied on each slide for 10 min and washed three times. After blocking (0.3% TritonX, 1% bovine serum albumin, and 5% normal donkey or goat serum) for 20 min, the slides were incubated with primary antibodies (Additional Table 1) overnight at 4 °C. The following day, slides were washed 3X with PBS and then incubated with secondary anti-rabbit/mouse/goat Alexa Fluor 488/546/647 (Invitrogen, Carlsbad, CA) antibodies for 2 h at room temperature in the dark. For Phalloidin or neurofilament (NF) staining, the secondary antibody was changed to anti-Phalloidin conjugated with Alexa Fluor 547 or 647 (1:200 in PBS), or anti-NF antibody conjugated with Alexa Fluor 555 (1:500 in PBS) for 30 min at room temperature. The slides were then washed with PBS and coverslipped with mounting medium with DAPI (Southern Biotech, Birmingham, AL or Vector, Burlingame, CA).

For whole-mount preparations, cochleae were dissected into the whole turns of organ of Corti under the microscope and stored at 4 °C in PBS until further processing. After three PBS washes, blocking solution was added (1% TritonX, 1% bovine serum albumin, and 10% normal donkey or goat serum) and tissue was incubated at room temperature (RT) for 1 h on a rotator. Next, blocking solution was exchanged with primary antibody solution (Additional Table 1) (0.3% TritonX, 1% bovine serum albumin, and 5% normal donkey or goat serum) overnight at 4 °C on the rotator. The next day, tissue was washed 3X with PBS and then incubated with secondary anti-rabbit/mouse/goat conjugated with Alexa Fluor 488/546/647 (Invitrogen, Carlsbad, CA) antibodies for 2 h at RT on the rotator protected from light. For Phalloidin or neurofilament (NF) staining, another 30 min RT incubation with anti-Phalloidin conjugated with Alexa Fluor 547 or 647 (1:200 in PBS), or anti-NF antibody conjugated with Alexa Fluor 555 (1:500 in PBS) was added after the secondary antibody incubation. The tissues were then washed with PBS and mounted on slides with DAPI containing mounting medium (Southern Biotech, Birmingham, AL or Vector, Burlingame, CA). For capturing images, we used light fluorescent filter microscope (Zeiss Axio Observer A1, Oberkochen, Germany) or confocal microscope (Olympus FV1000 FCS/RICS, oil obj. × 63, NA 1.4, Center Valley, PA).

### Cell count and statistical analyses

We defined transplanted human cells as those cells that express GFP (because LT2e hESCs line is expressing GFP constitutively) and are also positive for DAPI nuclear stain. We sampled the entire cochlea and analyzed all the sections by manually counting the number of those double-positive transplanted cells for both cross-sections and whole-mount tissue in each area. For the statistical analysis, we included only those mice in which we found any of GFP+/DAPI+ cells (Figs. 3 and 4B, C), we analyzed data statistically using the Student t test and ANOVA depending on the experimental design. Data are presented in the text and figures as means ± standard deviation (SD). The statistical significance level was set at *p* < 0.05.

### ABR measurements

Animals at P 27–P 30 (D 26–28) were anesthetized by intraperitoneal administration of xylazine (12.5 mg/kg) and ketamine-HCl (87.5 mg/kg) in saline. One-third dose of the anesthetic mix was administered as boosters every 30 min. Stimuli presentation and acquisition of evoked potentials were performed via an RME (Haimhausen, Germany) Hammerfall DSP Multiface II sound card (44.1 kHz sampling rate). Monaural auditory thresholds were assessed in response to 50 ms tone pips at frequencies of 4, 8, 16, and 22 kHz at stimulus levels from 20 to 95 dB SPL at 5-dB steps. Five hundred repetitions were presented for each ear; all conditions were randomized. Sound stimuli were delivered via custom insert earpieces attached to TDT (Tucker Davis Technologies, Inc., Alachua, FL) FF1 magnetic speakers. Evoked auditory potentials were recorded with platinum subdermal needle electrodes placed at the apex (active), and nape of the neck (reference) with a ground electrode placed in the hind leg. The threshold was defined as the lowest level at which any wave in the ABR could be clearly detected by visual inspection.

## Results

### Stem cell differentiation in vitro

**Fig. 1:**
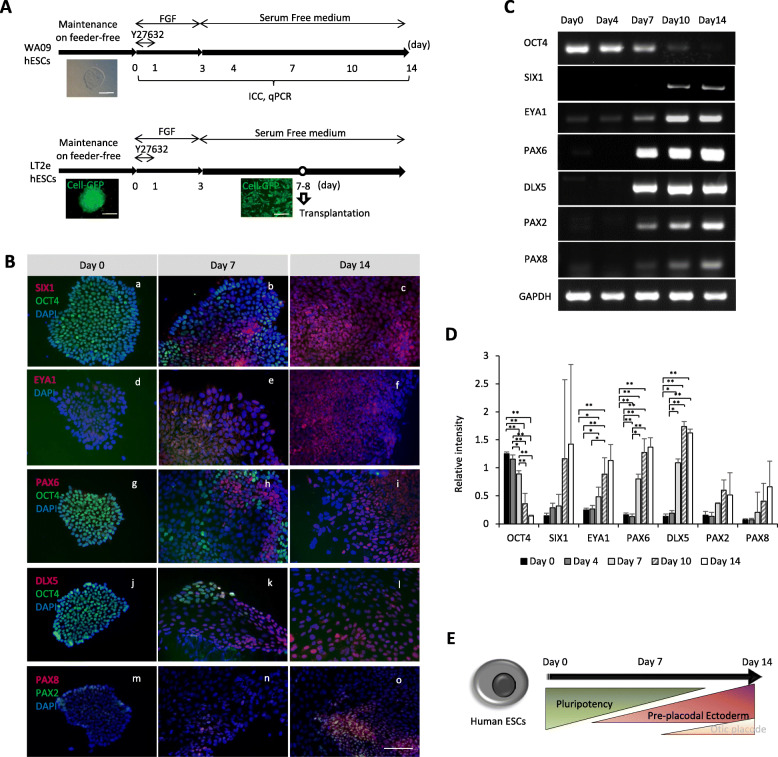
Protocols and results of stem cell differentiation in vitro. **A** A schematic illustrating cell cultures of WA09 and LT2e human ES cell lines in vitro. **B** Immunocytochemistry results at day 0 (**a,d,g,j**, and **m**), day 7 (**b, e, h, k**, and **n**), and day 14 (**c, f, I, l**, and **o**) of the WA09 cell line. SIX1 and OCT4 (**a–c**), EYA1 (**d–f**), PAX6 and OCT4 (**g–i**), DLX5 and OCT4 (**j–l**), and PAX2 and PAX8 staining (**m–o**) are shown. Pluripotent marker; OCT4 is expressed strongly on day 0, slightly on day 7, and not expressed on day 14. Pre-placodal ectoderm markers; SIX1, EYA1, and PAX6, and Dlx5 starts expressing at day 7 and continue to increase their expression as seen on day 14. Otic markers; PAX2 and PAX8 are slightly expressed on day 14. Scale bar represents 50 μm. **C** Representative semi-qPCR results showing the levels of each gene expression. **D** Quantification of the expression levels of each gene. OCT4 expression decreases, while PPE markers and OP markers increase with time of differentiation. Biological sample numbers were 3 and each sample was replicated twice. *P < 0.05; **P < 0.01. **E** Schematic shows our protocol to differentiate human ES cells in vitro. ES cell, embryonic stem cell; PPE, preplacodal ectoderm; OP, otic progenitor

WA09 human ES cells (hESCs) were differentiated for up to 14 days with Leung et al’s protocol [17] (Fig. 1A). Using immunocytochemistry, we detected the pluripotency marker OCT4 in almost all cells at day 0 (Fig. 1Ba, g, and j), and its positive labeling was present in a fraction of cells by day 7 but was no longer detected at day 14 (Fig. 1 Bc, i, and l). Immunostaining of the pre-placodal ectoderm (PPE) markers SIX1, EYA1, PAX6, and DLX5 was present at day 7 and over 50% of cells per well were positive for these markers at day 14 (Fig. 1Bc, f, and I; Additional Figure 2). On the other hand, immunolabeling of the otic placode (OP) markers PAX2 and PAX8 were seen only in a few cells at day 7 (Fig. 1Bn), and at a very low level at day 14 (Fig. 1Bo), where most cells were expressing either of these markers indeed expressed both (Fig. 1Bo). Next, we looked at each marker’s gene expression using semi-quantitative PCR (semi-qPCR). The relative intensity of Oct4 bands decreased gradually as cells differentiated (day 0 > day 4 > day 7> day 10 > day 14) (Fig. 1C, D). The markers of pre-placodal ectoderm EYA1 and PAX6, and DLX5 mRNA expression gradually increased from day 7 to day 14 (Fig. 1C, D), while another PPE marker SIX1 mRNA expression was not significant. Expression of OP markers, PAX2 and PAX8 mRNA, was not significantly raised up to day 14 though their bands were detectable at days 7, 10, and 14 (Fig. 1C, D). Taken together, pluripotency markers were downregulated at differentiation, PPE markers were upregulated with differentiation, and OP markers were partially upregulated (Fig. 1E). Therefore, we assume that the seventh day of differentiation represents cells at “early PPE-like” stage and the 14th day of differentiation represents cells at “late PPE-like” stage. In order to see differentiation patterns of LT2e hESCs, which were utilized for transplantation experiments, we performed semi-qPCR for LT2e cells as well as WA09. LT2e cells were differentiated using the same method as WA09 hESCs and had similar gene expression patterns to WA09 cells (Additional Figure 1).

### Effect of diphtheria toxin (DT) on neonatal mice

**Fig. 2:**
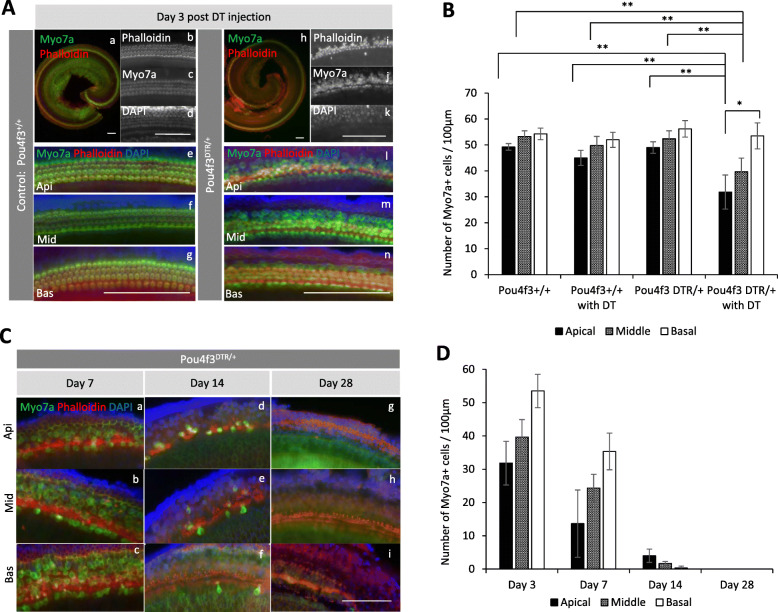
Immunohistological analysis of WT and Pou4f3DTR/+ mice after DT administration. **A** Immunohistochemistry of WT (Pou4f3+/+) and Pou4f3DTR/+ mouse organ of Corti at day 3 after DT administration. Green indicates Myo7a, red indicates Phalloidin, and blue indicates nuclear (DAPI) staining. Hair cells of WT mice are intact (**a**) for apical (**b–e**), middle (**f**), and basal turn (**g**), while those of Pou4f3DTR/+ mice, especially in the apical (**h–l**) and middle turn (**m**), lose their form. Scale bars represent 100 μm. **B** The graphs show numbers of Myo7a+ cells in the cochlear turns on day 3 after DT injection. The numbers in the apical and middle turn of Pou4f3DTR/+ mice are significantly lower than those of WT (*n* = 4), WT with DT (*n* = 5), and Pou4f3DTR/+ mice without DT (*n* = 5). In the Pou4f3DTR/+ with DT group (*n* = 6), the number of Myo7a+ cells in the apical turn is significantly higher than that in the basal turn. *P < 0.05. **P < 0.01 (one way ANOVA and Student’s t test). **C** Immunohistochemistry of Pou4f3DTR/+ mouse organ of Corti at day 7 (**a–c**), day 14 (**d–f**), and day 28 (**g–i**) after DT administration. Green indicates Myo7a, red indicates Phalloidin, and blue indicates nuclear (DAPI) staining. The number of Myo7a+ cells is small in all cochlear turns. Scale bars represent 50 μm. **D** The graphs show numbers of Myo7a+ cells in the cochlear turns on day 3, day 7, day 14, and day 28. The number of Myo7a+ cells in each cochlear turn of Pou4f3DTR/+ mice gradually decreases after DT injection. The biological sample number is 6 for day 3, 3 for days 7 and 14, and 4 for day 28. WT, wild type; DTR, diphtheria toxin receptor; DT, diphtheria toxin

First, we tested the effects of DT on WT mice cochlea since one previous report suggested that a large amount (50 ng/g body weight) of DT causes hearing loss in C57BL/6 WT mice [23]. Since in our protocol a dose of 2–4 ng/g has been used to ablate hair cells in Pou4f3DTR/+ mice, we tested this dosage in the WT C57BL/6 and found that at 3 days after the DT administration all hair cells (HCs) in the entire OC were intact. The hair cells had typical morphology as revealed by Myo7a immunolabeling, and phalloidin-stained stereocilia were lined up correctly (Fig. 2Aa–g), and also, there was no significant difference in the number of hair cells between non-treated WT mice and DT-treated WT mice (Fig. 2B). Furthermore, at 26–28 days after DT injection, the morphology of the organ of Corti (OC) was still intact (Fig. 5Ba) and hearing threshold was not decreased in WT mice (Additional Figure 3). These results suggest that 2–4 ng/g DT did not affect the number of hair cells and did not alter the hearing of WT neonatal mice.

Next, we assessed the effectiveness of DT on Pou4f3DTR/+ mice at various time points. Diphtheria toxin binds DT receptors only in Pou4f3-expressing HCs in the inner ear and ablates them. At 3 days after treatment (this time point reflects the day of cell transplantation), Myo7a–positive (Myo7a+) HCs and Phalloidin+ stereocilia started to change morphology especially in the apical and middle turn (Fig. 2 Ah–n), and the number of Myo7a+ cells in the apical and middle turn was significantly decreased when compared with WT, WT with DT, and Pou4f3DTR/+ without DT (Fig. 2B), while the basal turn Myo7a+ cells were almost intact at this time point. The number of Myo7a+ cells in the apical turn was significantly lower than in the basal turn (Fig. 2B). The number of HCs in the Pou4f3DTR/+ mice gradually decreased until no Myo7a+ cells could be found in any of the cochlear turns by the 28th-day post-DT injection (Fig. 2C, D). Also, ABR waveforms were not detectable from the 26th–28th day after treatment (Additional Figure 6). These data show that DT in early postnatal ages completely ablated all the HCs and within a month induced complete deafness in Pou4f3DTR/+ mice.

### Cell transplantation into WT mice

**Fig. 3:**
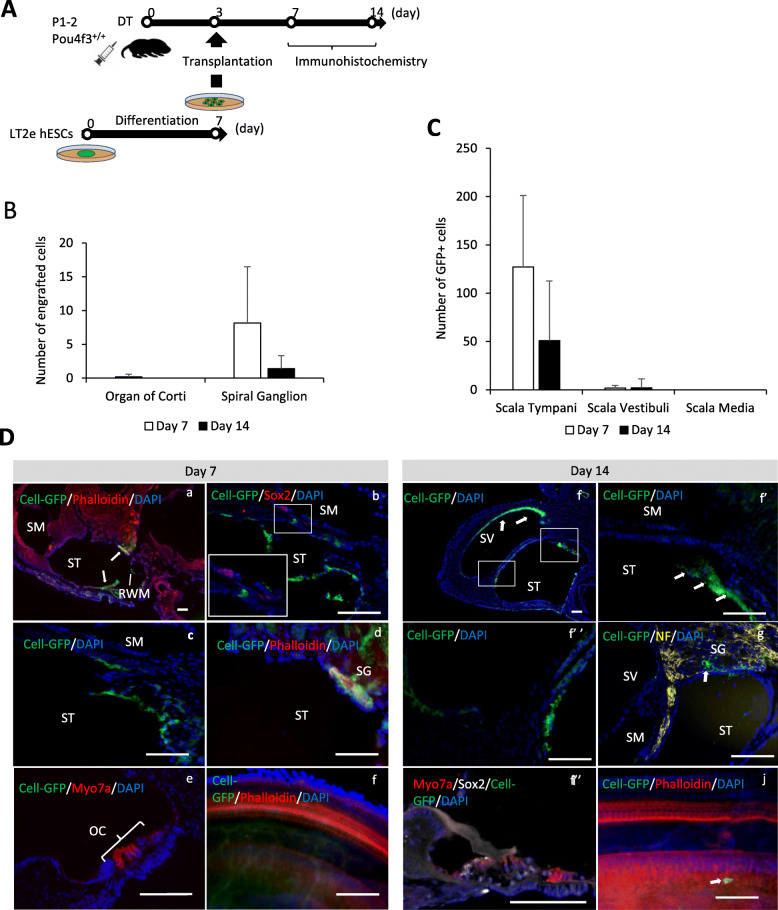
Immunohistological and cell count results of WT mice on day seven and day 14 after cell transplantation. **A** Schematic of the protocol of cell transplantation. **B** The graph shows the numbers of transplanted human cells in the organ of Corti and the spiral ganglion of the mouse cochlea at day 7 and day 14 after transplantation, *n* = 6 for day 7 and *n* = 4 for day 14. **C** The graph shows numbers of transplanted human cells in the scala tympani, scala vestibuli, and scala media of the mouse cochlea on day 7 and day 14 after transplantation, *n* = 6 for day 7 and *n* = 4 for day 14. **D** Immunohistochemistry of the cochleae at day 7, day 14 after cell transplantation. Green indicates Cell-EGFP, White indicates Sox2, yellow indicates neurofilament, and blue indicates DAPI. Red staining includes Myo7a, Phalloidin, and Sox2. Most of the transplanted cells are in the scala tympani. No cells were found in the organ of Corti, while a few cells migrated in the spiral ganglion. White arrows show the transplanted cells. Scale bars represent 100 μm. SM, scala media; ST, scala tymoani; RWM, round window membrane; SV, scala vestibule; WT, wild type

Three days after DT injection at postnatal days 1–2 (P1–2), we transplanted 7-day-differentiated hESCs (equivalent to the early PPE-like stage) into P4–5 WT mouse perilymph (Fig. 3A). The cells were identified in the mouse cochlea as transplanted if they expressed GFP and stained with DAPI. At day 7 (4 days after transplantation), 127.2 ± 74.1 transplanted human cells were found in the scala tympani; most of them were floating and some of them seemed attached to the epithelium (Fig. 3B, Da–d). In the scala vestibuli, there were 1.8 ± 2.8 human cells and no cells were found in the scala media (Fig. 3B). Although one human cell looked like it was migrating into the organ of Corti, it was still in the basilar membrane (Fig. 3C, Db). Altogether, on day 7, we did not detect any hES cells in the organ of Corti epithelium (Fig. 3De and f); however, 8.2 ± 8.3 human cells had migrated into the spiral ganglion (Fig. 3C, Dd). At day 14 (11 days after transplantation), the number of transplanted human cells in the scala tympani decreased to 51 ± 61.7 from 127.2 ± 74.1 at day 7, and a few cells still remained in the scala vestibuli (Fig. 3B, Df). Most of the cells in the scala tympani were floating (Fig. 3Df’ and Df”). We found no human cells in the scala media at day 7 nor day 14 (Fig. 3B); similarly, no human cells were detected in the organ of Corti (Fig. 3C, Di, and Dj), and few human cells (3.5 ± 1.9) were engrafted in the spiral ganglion when compared to day 7 (Fig. 3C, Dg, and Dj). These data show that human cells transplanted to WT mouse cochlea did not migrate into the scala media or organ of Corti epithelium, even though some of the cells survived in the perilymph for up to 11 days. However, a few human cells migrated/engrafted into the spiral ganglion. At days 26–28 (23–25 days after transplantation), no cells could be found in the scala tympani or in the organ of Corti (Fig. 5Ba, C). Auditory brainstem response thresholds revealed that the cell transplantation procedure itself did cause slight hearing loss (Figure S5). Of note, we did not use any immunosuppression and did not find teratoma formation at any time point.

### Transplantation into the hair cell ablated cochlea

**Fig. 4:**
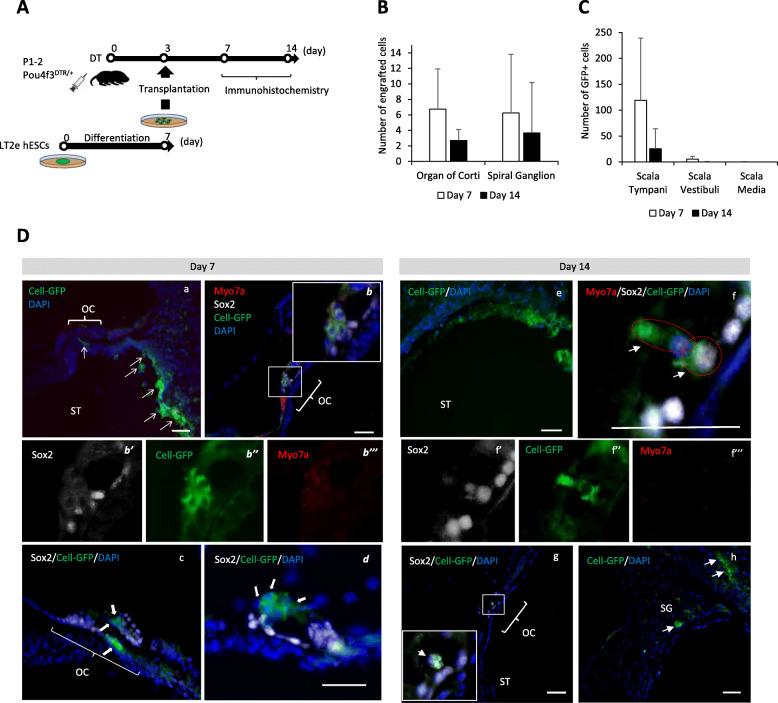
Immunohistological and cell count results of Pou4f3DTR/+ mice at day 7 and day 14 after cell transplantation. **A** Schematic of the protocol of cell transplantation. **B** The graph shows the numbers of transplanted human cells in the organ of Corti and the spiral ganglion of the mouse cochlea at day 7 and day 14 after transplantation. *n* = 4 for day 7 and *n* = 3 for day 14. **C** The graph shows numbers of transplanted human cells in the scala tympani, scala vestibule, and scala media of the mouse cochlea at day 7 and day 14 after transplantation. *n* = 4 for day 7 and *n* = 3 for day 14. **D** Immunohistochemistry of the cochleae at day 7, day 14 after cell transplantation. Green indicates Cell-EGFP, white indicates Sox2, and blue indicates DAPI. Although most of the transplanted cells are in the scala tympani, some cells have migrated into the organ of Corti and express Sox2. Db’, b”, b”’, f’, f”, and f”’ show single channel for Db and Df, respectively. White arrows show the transplanted cells. Scale bars represent 50 μm. OC, organ of Corti; SG, spiral ganglion; DTR, diphtheria toxin receptor

Similar to WT mice, 7-day-differentiated human ESCs (early PPE-like stage) were transplanted into Pou4f3DTR/+ mice on day 3 after DT-induced specific HC ablation (Fig. 4A). At day 7, 123.3 ± 93 human transplanted cells were found mostly floating, in the scala tympani (Fig. 4C, Da), 5.8 ± 5.7 human cells in the scala vestibuli, and one cell was found in the scala media (Fig. 4C). In contrast to WT mice, 6.75 ± 4.5 human cells migrated into the epithelium of the organ of Corti, but they were found to be negative for either Sox2 or Myo7a at this time point (Fig. 4Db–d). Furthermore, 9.75 ± 9.5 human cells migrated into the spiral ganglion at day 7 (Fig. 4B).

At day 14, 23.3 ± 40.4 human cells remained, mostly floating in the scala tympani, and there were no cells found in the scala vestibuli or the scala media (Fig. 4C). We also found that 2.7 ± 1.5 human cells were engrafted in the organ of Corti (Fig. 4B), and some of these cells were stained with antibody anti- Sox2, but not with the anti-Myo7a antibody (Fig. 4Df and g). In the spiral ganglion, 5.3 ± 9.2 cells engrafted (Fig. 4B, Dh). Furthermore, we found few cells in the scala media. Based on these findings, we presume that the cells detected in the organ of Corti migrated through the basilar membrane directly from the scala tympani, avoiding the endolymphatic milieu of the scala media. Engrafted cells in the organ of Corti were observed in the basal and middle turns, not in the apical turn.

**Fig. 5:**
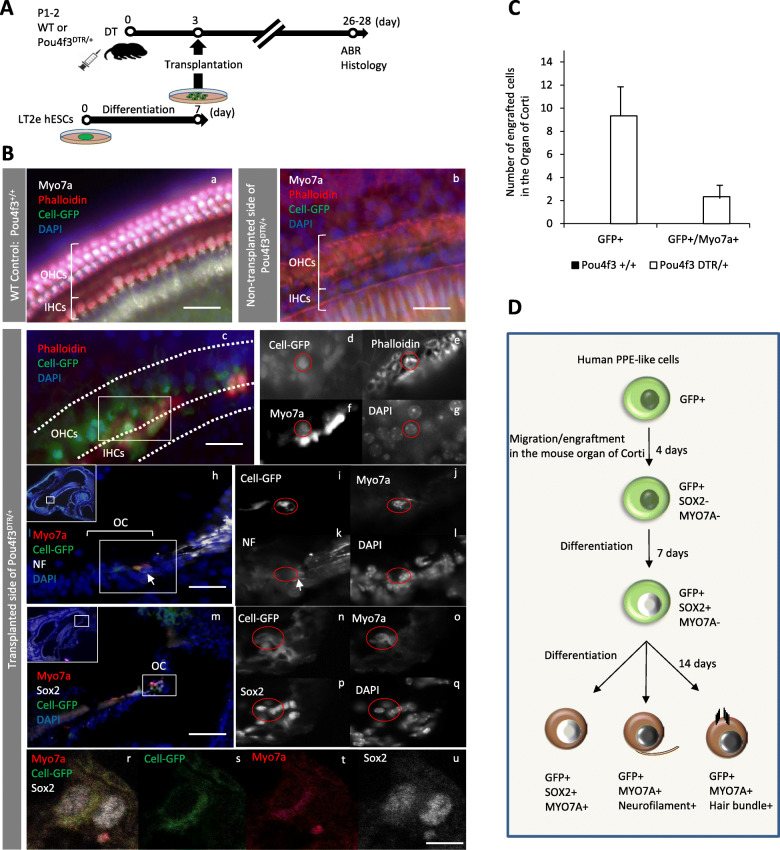
Immunohistological and cell count results of WT and Pou4f3DTR/+ mice at day 26-28 after cell transplantation. **A** Schematic of the protocol of cell transplantation. **B** Immunohistochemistry results at days 26–28 after cell transplantation. Green indicates Cell-EGFP, white indicates Myo7a or Sox2, red indicates Phalloidin or Myo7a, yellow indicates neurofilament-200 kDa, and blue indicates DAPI staining. The organ of Corti of WT mouse is intact but no GFP-positive cells were found. There is neither hair cell nor transplanted human cell in the organ of Corti of non-transplanted side of Pou4f3DTR/+ mouse. There are some GFP+ cells in the transplanted side of Pou4f3DTR/+ mouse, and some of them are positive for Sox2, Myo7a, and NF. White arrow (h) shows the neurofilament (NF) toward the engrafted cells. Scale bars represent 50 μm for c, h, and i, 10 μm for u. **C** The graph shows numbers of engrafted cells in the organ of Corti of the WT and Pou4f3DTR/+ mouse cochleae at day 28 after transplantation. *n* = 3. **D** Schematic of the differentiation of the transplanted human cells in vivo. OHCs, outer hair cells; IHCs, inner hair cells; OC, organ of Corti; WT, wild type; DTR, diphtheria toxin receptor

At days 26–28 (Fig. 5A), 9.3 ± 7.5 engrafted cells were found in the basal and 1.67±1.52 in the middle turn, and 0 ± 0 in the apical turn of the OC (each *n* = 3) (Fig. 5Bc–u, C). 2.3 ± 2.5 (approximately 25%) of those cells in the basal turn were labeled with anti-Myo7a antibody (Fig. 5Bh–u, C). Furthermore, one of the engrafted human cells in the mouse OC had hair bundles (Fig. 5Bc–g, D), and some of the engrafted cells labeled not only with anti-Myo7a but also anti-Sox2 antibodies (Fig. 5Bm–u) Neuro-filament antibody-positive neuronal fibers were also found in the vicinity of a GFP+/Myo7a+ cell (Fig. 5Bh–l). From one frozen section, 50% of the engrafted human cells were positive for only Sox2 and 30% immunolabeled with both Sox2 and Myo7a in the mouse OC (Figure S4). In summary, these results suggest that the transplanted PPE-like human cells could engraft in the hair cell ablated mouse OC and some of the engrafted cells were able to differentiate into inner ear hair cells through a Sox2-positive stage within a month in vivo. Additionally, the neuronal fibers present in the vicinity of the engrafted hair cells (Fig. 5D) suggest that the engrafted cells might attract the peripheral processes of spiral ganglion neurons. Moreover, a few human cells were also detected in the spiral ganglion and they immunolabeled with neuronal markers: MAP 2 and NF-200 kDa on days 26–28 (Additional Figure 8). Nevertheless, hearing thresholds were not improved with transplantation (Figure S6). Similar to our WT mouse experiments, we did not use any immunosuppression and did not observe teratoma formation at any time point.

## Discussion

We were motivated by two primary concerns when deciding the stage of differentiation of the cells to be used for the transplantation experiments. First, we wanted to avoid teratoma growth, which usually forms from undifferentiated stem cells. Secondly, we presumed that grafting cells at an early, rather than late, stage of differentiation would have increased chances of their successful migration from the perilymph through the basilar membrane into the organ of Corti. We decided that the pre-placodal ectoderm stage of differentiation might fulfill both of those requirements, and indeed we did not see any teratoma formation in either the WT and transgenic mice. We used a previously described PPE differentiation protocol based on serum-free culture conditions [17]. We have observed that the pluripotency marker OCT4 is present in the culture at day 7 but disappears completely by day 14. At the same day 7 PPE markers (SIX1, EYA1, PAX6, DLX5) increase expression, and at day 10 there is a gradual increase of otic markers (PAX2, PAX8) expression levels. Considering the measured increase in the expression of otic markers, this protocol likely could have been modified with key molecules (BMP4, BMP blocker, or Wnt activator, etc) for more efficient otic differentiation [8]. Taken together, we conclude that we achieved direct human stem cell differentiation through the early PPE-like cells by day seven and toward a late PPE-like stage by day 14 of culture.

Lang and colleagues showed that the injured, rather than the intact sensory epithelium of the inner ear, might create the environment more conducive to engraftment of transplanted cells [18]. We, therefore utilized a Pou4f3+/DTR mouse with diphtheria toxin injection to induce hair cell death. This model allows for very selective elimination of sensory cells but leaves non-sensory epithelium intact [19]. Comparing the effects DT has on the sensory epithelium we showed that no hair cell death is observed 3 days post-injection in the WT mice while in the Pou4f3+/DTR the earliest signs of hair cell ablation are already detectable. Furthermore, in the WT mouse 26–28 days post-DT injection the morphology of the organ of Corti (OC) was still intact (Fig. 5Ba) and hearing thresholds were normal (Additional Figure 3). In contrast, 28 days post-DT injection the Pou4f3+/DTR mouse sensory epithelium along the entire cochlear frequency range was ablated, and the ABR thresholds increased up to 90 dB SPL (Figure S2). These experiments show that DT, used at the concentration of 2–4 ng/g, injected into the WT mouse is not sufficient to induce morphologically detectable damage to the sensory epithelium, and it does not impact ABR thresholds. As such, we found that the Pou4f3+/DTR mouse is an excellent model for acute and severe ablation of hair cells in the organ of Corti.

In our transplantation experiments, the majority of injected cells were found floating in the scala tympani at 7 days post-DT injection (over 100 cells), and by day 14 we observed a 3- to 5-fold decrease in the number of these floating cells in both WT and Pou4f3+/DTR mice. Seven days after cell injection, only a few cells were found in the scala vestibuli or scala media, and a small number (5-10 cells) were detected in the spiral ganglion of both WT and Pou4f3+/DTR ablated mice. Importantly, we have observed migration of larger number of cells in the OC when injected in the transgenic mice whose HCs had been ablated than in the intact OC of the WT mice. In both cases, the number of migrated cells was smaller in OC and SG two weeks after injection as compared to the number of cells found at 1-week post-injection. Cells in the scala tympani remained floating up to 14 days later. Furthermore, no floating cells were found in the scala media, an area to which cells could get to by crossing the basilar membrane. Therefore, we suspect that the cells that did cross the basilar membrane probably migrated directly into the organ of Corti from the scala tympani. Out of the engrafted cells found in the organ of Corti and spiral ganglion seven days after their injection only a fraction were present at 14 days. This suggests that the engrafted cells did not survive in their new environment, possibly due to insufficient blood supply, apopotisis or immune system rejection. Nevertheless, here we have demonstrated that human stem cells in vitro differentiated to the early PPE stage can engraft into ablated mouse organ of Corti and form hair cell-like cells, as identified by immunohistochemical methods.

We found a relatively low number of engrafted cells that differentiated into hair-cell-like cells, and that more cells engrafted in the injured rather than healthy organ of Corti. Conversely, we saw no difference between the number of floating cells and the number of cells engrafted in the spiral ganglion between transgenic and wild type mice cochleae. This indicates that the injured organ of Corti has no influence on cell survival in other regions of the cochlea. However, it indeed might promote engrafted cell survival in the organ of Corti, where the hair cell ablation was specifically induced. Our observation agrees with previous reports where pharmacologically ablated organ of Corti promoted better cellular engraftment and survival [18]. The results of our experiments suggest that cell transplantation-based regenerative therapies for hearing loss might be achievable, but parameters such as the stage of differentiation of grafted cells, the type and severity of injury, the site of transplantation, the number of cells injected, and the potential use of immune suppression will all have to be evaluated in detail prior to realizing any promise of this approach in the future. In terms of site of transplantation, directly delivering into the scala media with a specific condition, which is reported by Lee and colleagues [24], might be an alternative method for better engraftment in the organ of Corti. In addition, Lopez et al. [12] recently showed engraftment of human iPS cell-derived otic progenitors in the adult guinea pig organ of Corti after amikacin-related deafening. They transplanted human iPS and Atoh1-GPF transgenic mouse-derived otic progenitor cells injected directly into the scala tympani similar to our approach. Their use of aminoglycoside for lesioning likely would be expected to lead to a less complete hair cell lesioning than our genetic lesioning strategy. In addition, they utilized a cochleostomy approach and we used a round window membrane approach though likely this would not have created significant differences in outcome given that both approaches led to injection into the scala tympani fluid space. Importantly, they utilized immunosuppression after xenografting whereas we did not in our studies. They concluded that injury to the organ of Corti promotes engraftment in the adult guinea pig, similar to our findings in the neonatal mouse. Given their findings, one wonders if the use of immunosuppression in our studies would have led to more robust survival and engraftment of transplanted cells, a topic we seek to investigate in future studies.

We used a GFP+ cell line to detect human cells in the mouse tissue. But auto-fluorescence in the tissue makes it hard to detect GFP+ cells, especially in adult tissue (Additional Figure 7). For enhancing the signal, we tried to use an anti-GFP antibody to no avail. As such, we might have missed some low GFP+ signal of transplanted human cells. On the other hand, we might have miscounted some mouse cells with auto-fluorescence as human cells although it seems less likely because there were no clear GFP+ cells in the non-transplanted side cochlea. One way of circumventing the confusion of signal source, i.e., GFP+ signal vs intense background induced by 488 nm (GFP) excitation would be to use immunolabeling of human-specific antigens, for example, the anti-human nucleus antibody STEM101 in future studies [11].

Some engrafted cells (GFP+) expressed both Myo7a and Sox2, an immunohistochemical indicative of a newly generated immature hair cell-like cell, and we showed that one of them had hair bundles and one of them had a neuronal fiber in close vicinity, which might suggest that some of those transplanted cells may be maturing to the functional stage. Future experiments will be needed to establish the transplanted cells’ identity and function at the later stages of maturation after transplantation.

We have demonstrated the feasibility of human stem cell engraftment into an ablated mouse organ of Corti. Our work suggests that there might be mechanisms, especially in the injured mouse organ of Corti that could permit/promote human cell differentiation toward the sensory cell fate after transplantation. Furthermore, these results shed light on future stem cell therapy of acute hearing loss in humans. Our findings of a low level of successful cellular engraftment, survival, and differentiation suggest that tailored induction and likely immune response control will be necessary for achieving a meaningful level of regeneration for restoration of hearing in the future.

## Conclusion

This study addresses whether human stem cells-derived progenitors can engraft in the mouse auditory epithelium after lesioning. We differentiated human pluripotent stem cells to the pre-placodal stage in vitro and delivered the cells to the mouse cochlea after selective hair cell lesioning. We found that such prepared cells engraft in the sensory epithelium and differentiate to hair and supporting cell immunophenotypes.

## Supplementary Information

**Additional file 1: Figure S1.** Results of semi-qPCR for LT2e cell line. The intensity of each band was quantified. The expression pattern of each gene is similar to that of the WA09 cell line. *n*= 3. **Figure S2.** Results of cell counts for immunocytochemistry. The number of each marker positive cells was quantified. The expression pattern of each protein is similar to that of mRNA. *n*= 1. **Figure S3.** ABR results at day 28 for WT and Pou4f3DTR/+ mice with or without DT. A hearing threshold of Pou4f3DTR/+ mice that received DT is increased over 90dB while hearing of WT with or without DT and Pou4f3DTR/+ without DT mice are preserved. *n*=4 for WT without DT, *n*= 6 for WT with DT, *n*= 4 for Pou4f3DTR/+ mice without DT and *n*=5 for Pou4f3DTR/+ with DT. WT; wild type, DTR; diphtheria toxin receptor, DT; diphtheria toxin. **Figure S4.** Results of cell viability tests. (A) The cell survival rate was assessed after ejecting from the micro-glass pipettes. Cells were completely clogged in the pipette when 12.5 μm sized tip was chosen, while 40-50% of cells were alive when 18.75 μm used. The best survival rate (90-100%) was recorded by 25 μm of tip. *n*=1 (B)(C) Cell viability test was performed with two different media, DMEM and SF media (B) or different temperature (C) using trypan blue solution in vitro (Thermo Fisher). SF media shows better survivability at 60- 120 mins after cell dissociation than DMEM. There is no difference between each temperature condition. Each *n*=1. DMEM; Dulbecco's Modified Eagle Medium. **Figure S5.** The graph showing the percentage of engrafted cells expressed supporting cell marker (Sox2) and hair cell marker (Myoa7a) in the OC at day 28 in a Pou4f3DTR/+ mouse after DT and cell transplantation. 50% of the engrafted cells were positive for Sox2 and 30% of the engrafted cells were Sox2 and Myo7a double-positive, while 20% of the engrafted cells did not express either Sox2 or Myo7a. OC; organ of Corti, DTR; diphtheria toxin receptor, DT; diphtheria toxin. **Figure S6.** ABR results at day 26-28 for WT and Pou4f3DTR/+ mice after cell transplantation. Hearing thresholds of Pou4f3DTR/+ mice with DT was not improved by cell transplantation. WT mice received both DT injection and cell transplantation shows slight damage to hearing. WT; wild type, DTR; diphtheria toxin receptor, DT; diphtheria toxin. **Figure S7.** Images of the organ of Corti at day 26-28 after cell transplantation in transgenic mice. A; non-transplanted side of the transgenic mouse cochlea, B; transplanted side of the transgenic mouse cochlea. A’ and B’ shows large images of square part in each image. Green indicates autofluorescence (thin arrow) and engrafted human cells (thick arrow). Red indicates phalloining staining. Bars represent 50 μm. **Figure S8.** Images of the spiral ganglion at day 14 and day 28 after cell transplantation in transgenic mice. Green indicates engrafted human cells in the mouse spiral ganglion. On day 14, some engrafted cells make cluster and those are Sox2 positive (a, b). At day 28, a very few cells are still engrafted in the spiral ganglion (c) and modiolus (d). Green; naïve GFP signal, white; Sox2, red; MAP2 or Tuj1, yellow; NF-200 and Blue; DAPI staining. Bars represent 50 μm. **Table S1.** Details on antibodies. **Table S2.** Primer sequences utilized.

## Data Availability

The datasets used and/or analyzed during the current study are available from the corresponding author on reasonable request.

## Abbreviations

HC(s): Hair cell(s)
OC: Organ of Corti
hESC: Human embryonic stem cells
PPE: Pre-placodal ectoderm
RW: Round window
RWM: Round window membrane
WT: Wild type
GFP: Green fluorescent protein
RT: Room temperature
NF: Neurofilament
DT: Diphtheria toxin
ST: Scala tympani
SV: Scala vestibule
