# Supplementary material for: Selective ablation of cochlear hair cells promotes engraftment of human embryonic stem cell-derived progenitors in the mouse organ of Corti
Source: Stem Cell Res Ther. 2021 Jun 19;12:352. doi: 10.1186/s13287-021-02403-9 (PMC8214253; doi:10.1186/s13287-021-02403-9)
Supplement: Supplementary file 1 — Additional file 1: Figure S1. Results of semi-qPCR for LT2e cell line. The intensity of each band was quantified. The expression pattern of each gene is similar to that of the WA09 cell line. n= 3. Figure S2. Results of cell counts for immunocytochemistry. The number of each marker positive cells was quantified. The expression pattern of each protein is similar to that of mRNA. n= 1. Figure S3. ABR results at day 28 for WT and Pou4f3DTR/+ mice with or without DT. A hearing threshold of Pou4f3DTR/+ mice that received DT is increased over 90dB while hearing of WT with or without DT and Pou4f3DTR/+ without DT mice are preserved. n=4 for WT without DT, n= 6 for WT with DT, n= 4 for Pou4f3DTR/+ mice without DT and n=5 for Pou4f3DTR/+ with DT. WT; wild type, DTR; diphtheria toxin receptor, DT; diphtheria toxin. Figure S4. Results of cell viability tests. (A) The cell survival rate was assessed after ejecting from the micro-glass pipettes. Cells were completely clogged in the pipette when 12.5 μm sized tip was chosen, while 40-50% of cells were alive when 18.75 μm used. The best survival rate (90-100%) was recorded by 25 μm of tip. n=1 (B)(C) Cell viability test was performed with two different media, DMEM and SF media (B) or different temperature (C) using trypan blue solution in vitro (Thermo Fisher). SF media shows better survivability at 60- 120 mins after cell dissociation than DMEM. There is no difference between each temperature condition. Each n=1. DMEM; Dulbecco's Modified Eagle Medium. Figure S5. The graph showing the percentage of engrafted cells expressed supporting cell marker (Sox2) and hair cell marker (Myoa7a) in the OC at day 28 in a Pou4f3DTR/+ mouse after DT and cell transplantation. 50% of the engrafted cells were positive for Sox2 and 30% of the engrafted cells were Sox2 and Myo7a double-positive, while 20% of the engrafted cells did not express either Sox2 or Myo7a. OC; organ of Corti, DTR; diphtheria toxin receptor, DT; diphtheria toxin. Figure S6. A [file 13287_2021_2403_MOESM1_ESM.docx]

**Additional Figures**


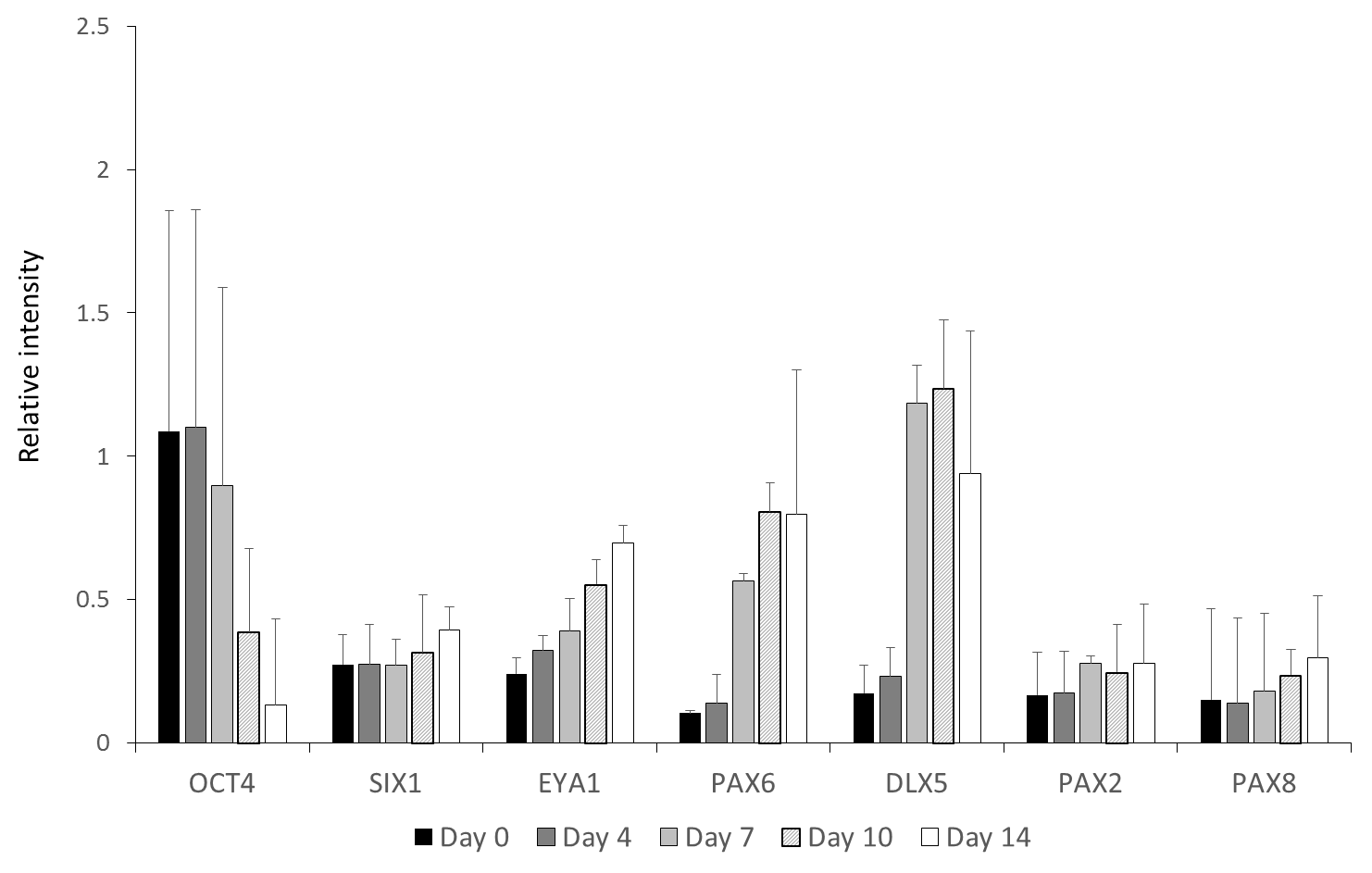


Additional Figure 1. Results of semi-qPCR for LT2e cell line. The intensity of each band was quantified. The expression pattern of each gene is similar to that of the WA09 cell line. n= 3.


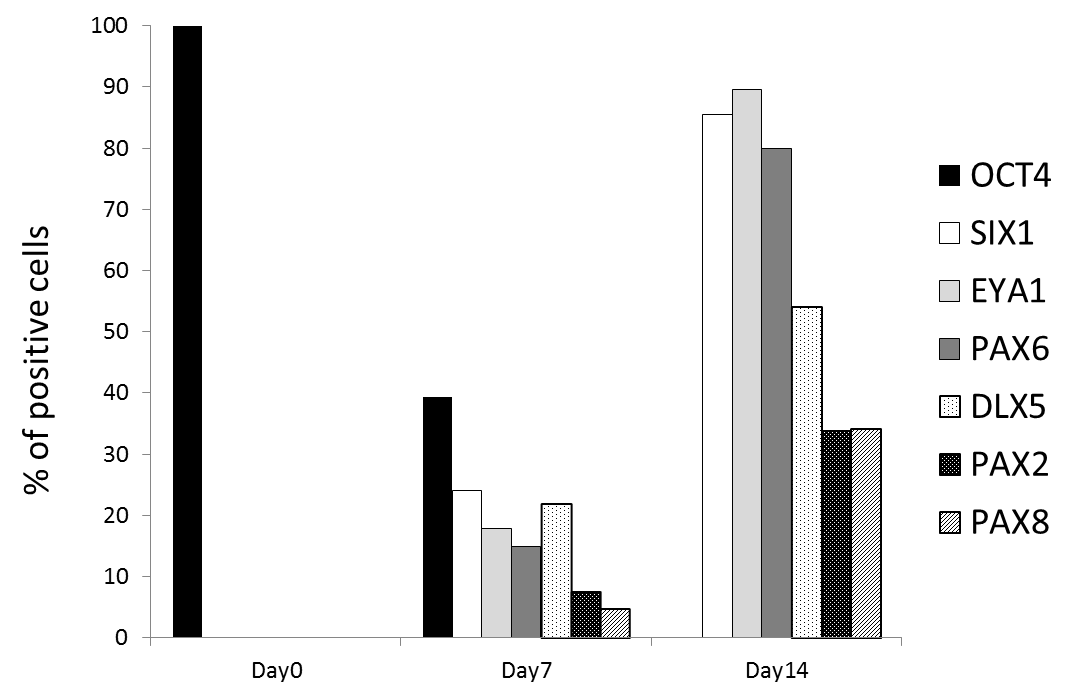


Additional Figure 2. Results of cell counts for immunocytochemistry. The number of each marker positive cells was quantified. The expression pattern of each protein is similar to that of mRNA. n= 1.

Method; the number of cell counts was determined by Nucleus Counter plug-in for ImageJ using the following setting: particle sizes ranged from 25 to 700 arbitrary units, background subtracted and watershed filtered. A total of 500–1000cells was counted for each marker.


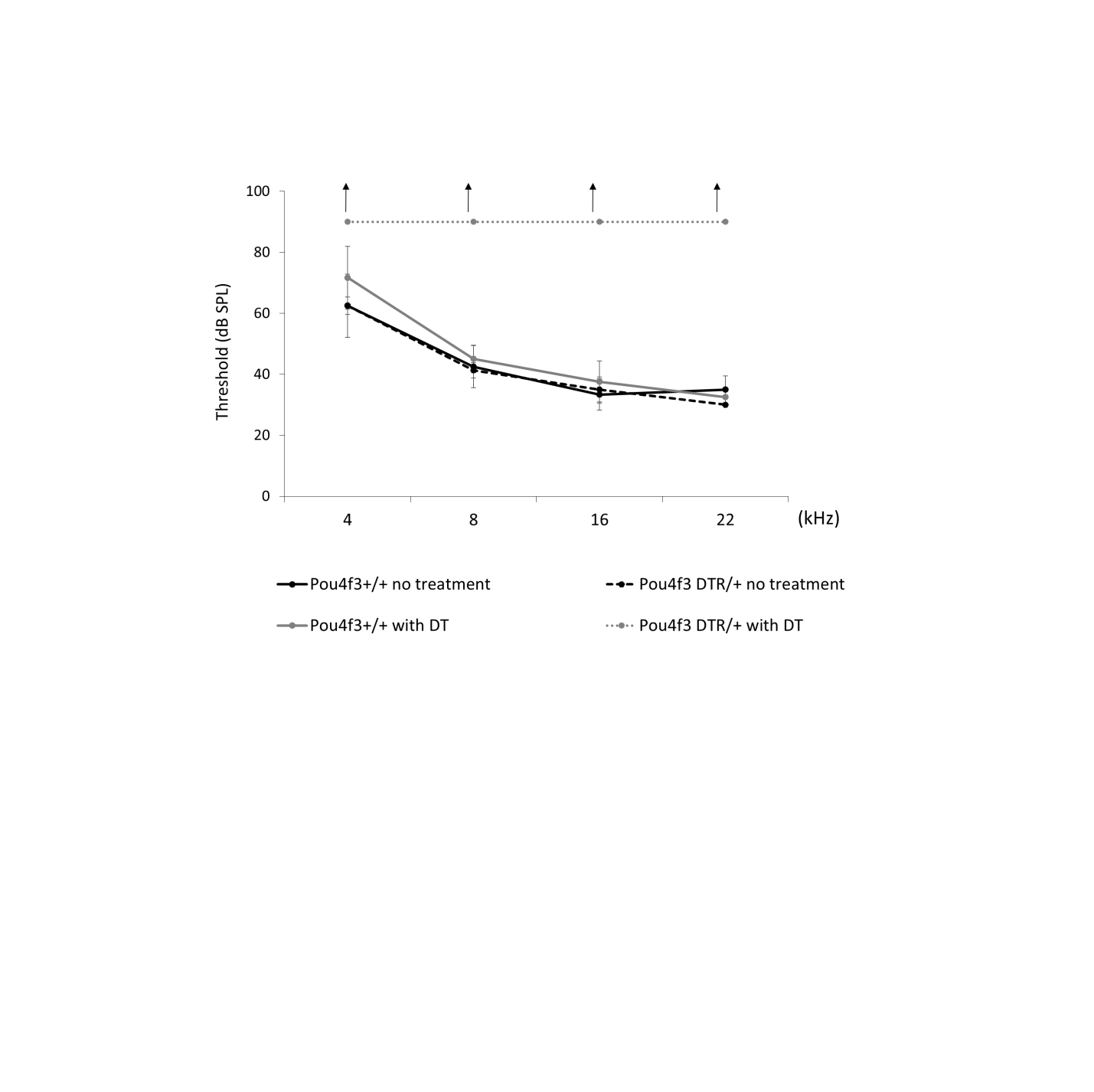


Additional figure 3. ABR results at day 28 for WT and Pou4f3DTR/+ mice with or without DT. A hearing threshold of Pou4f3DTR/+ mice that received DT is increased over 90dB while hearing of WT with or without DT and Pou4f3DTR/+ without DT mice are preserved. n=4 for WT without DT, n= 6 for WT with DT, n= 4 for Pou4f3DTR/+ mice without DT and n=5 for Pou4f3DTR/+ with DT. WT; wild type, DTR; diphtheria toxin receptor, DT; diphtheria toxin.


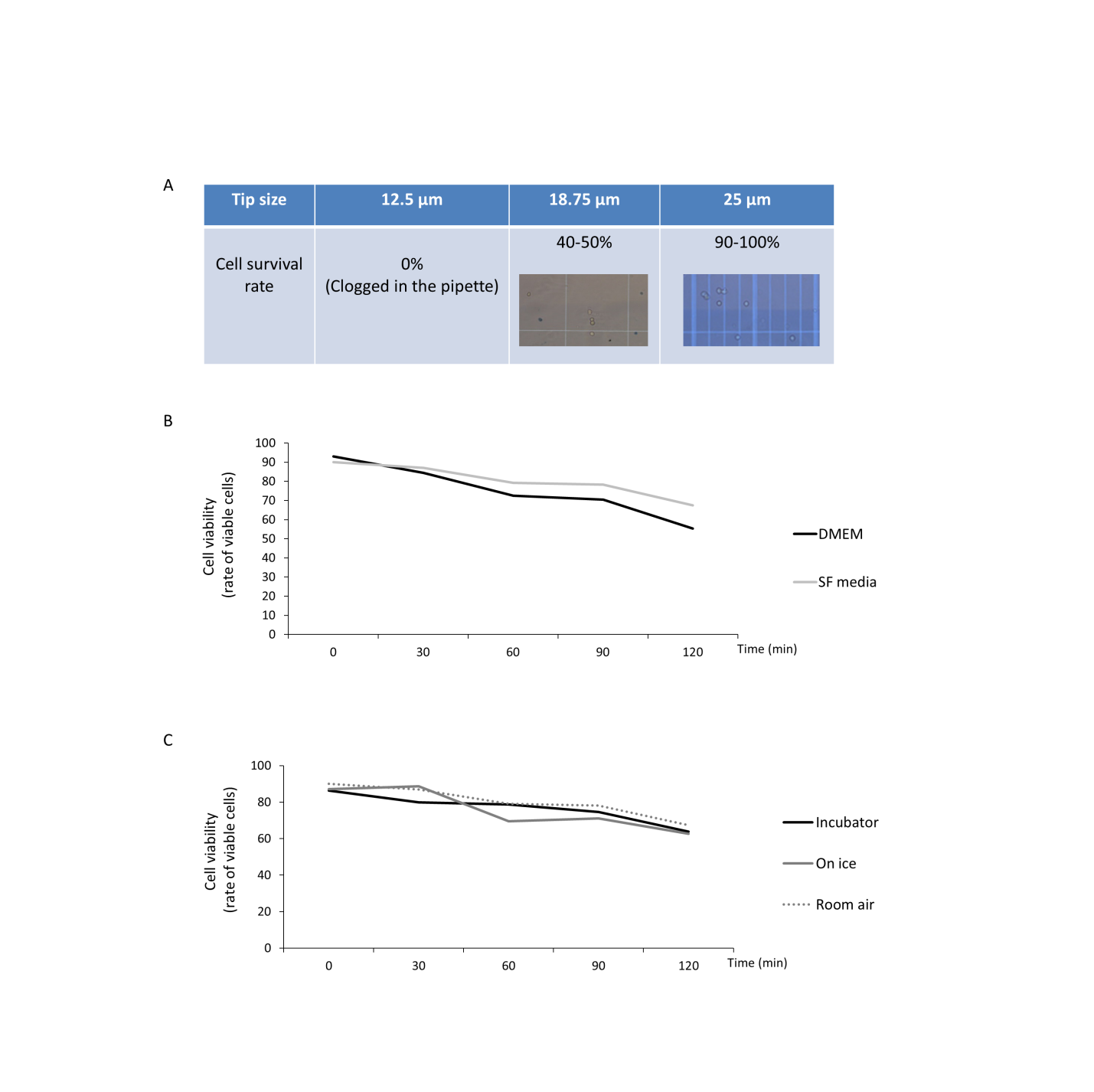


Additional figure 4. Results of cell viability tests. (A) The cell survival rate was assessed after ejecting from the micro-glass pipettes. Cells were completely clogged in the pipette when 12.5 μm sized tip was chosen, while 40-50% of cells were alive when 18.75 μm used. The best survival rate (90-100%) was recorded by 25 μm of tip. n=1 (B)(C) Cell viability test was performed with two different media, DMEM and SF media (B) or different temperature (C) using trypan blue solution in vitro (Thermo Fisher). SF media shows better survivability at 60- 120 mins after cell dissociation than DMEM. There is no difference between each temperature condition. Each n=1. DMEM; Dulbecco's Modified Eagle Medium.


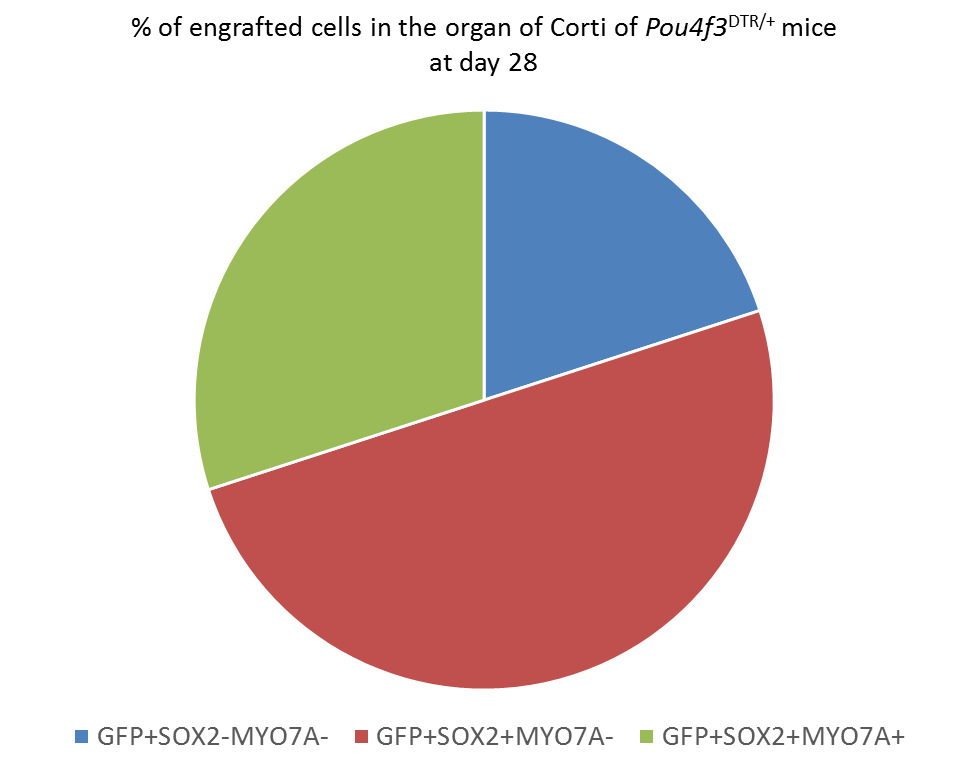


Additional figure 5. The graph showing the percentage of engrafted cells expressed supporting cell marker (Sox2) and hair cell marker (Myoa7a) in the OC at day 28 in a Pou4f3DTR/+ mouse after DT and cell transplantation. 50% of the engrafted cells were positive for Sox2 and 30% of the engrafted cells were Sox2 and Myo7a double-positive, while 20% of the engrafted cells did not express either Sox2 or Myo7a. OC; organ of Corti, DTR; diphtheria toxin receptor, DT; diphtheria toxin.


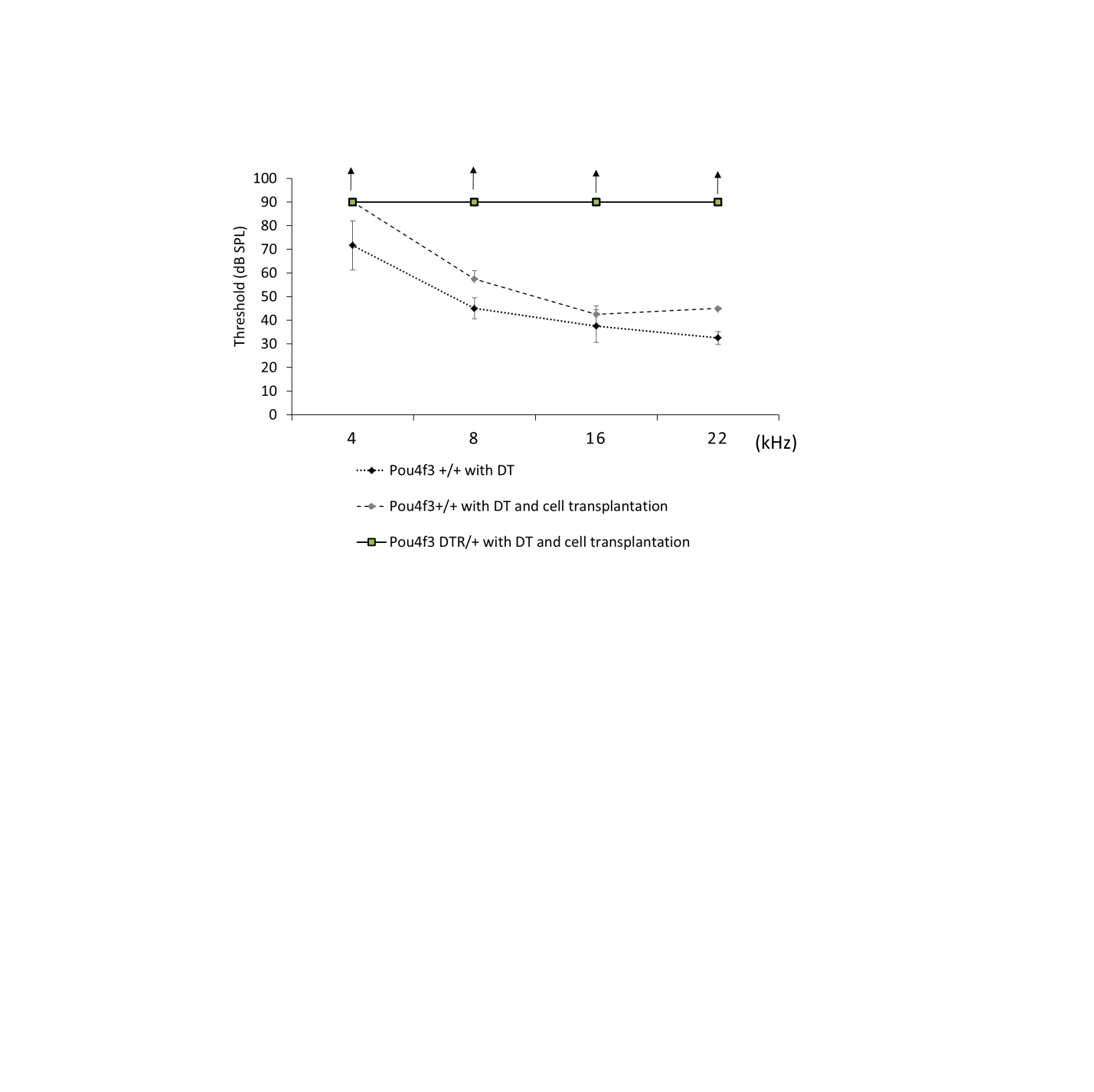
Additional figure 6. ABR results at day 26-28 for WT and Pou4f3DTR/+ mice after cell transplantation. Hearing thresholds of Pou4f3DTR/+ mice with DT was not improved by cell transplantation. WT mice received both DT injection and cell transplantation shows slight damage to hearing. WT; wild type, DTR; diphtheria toxin receptor, DT; diphtheria toxin.


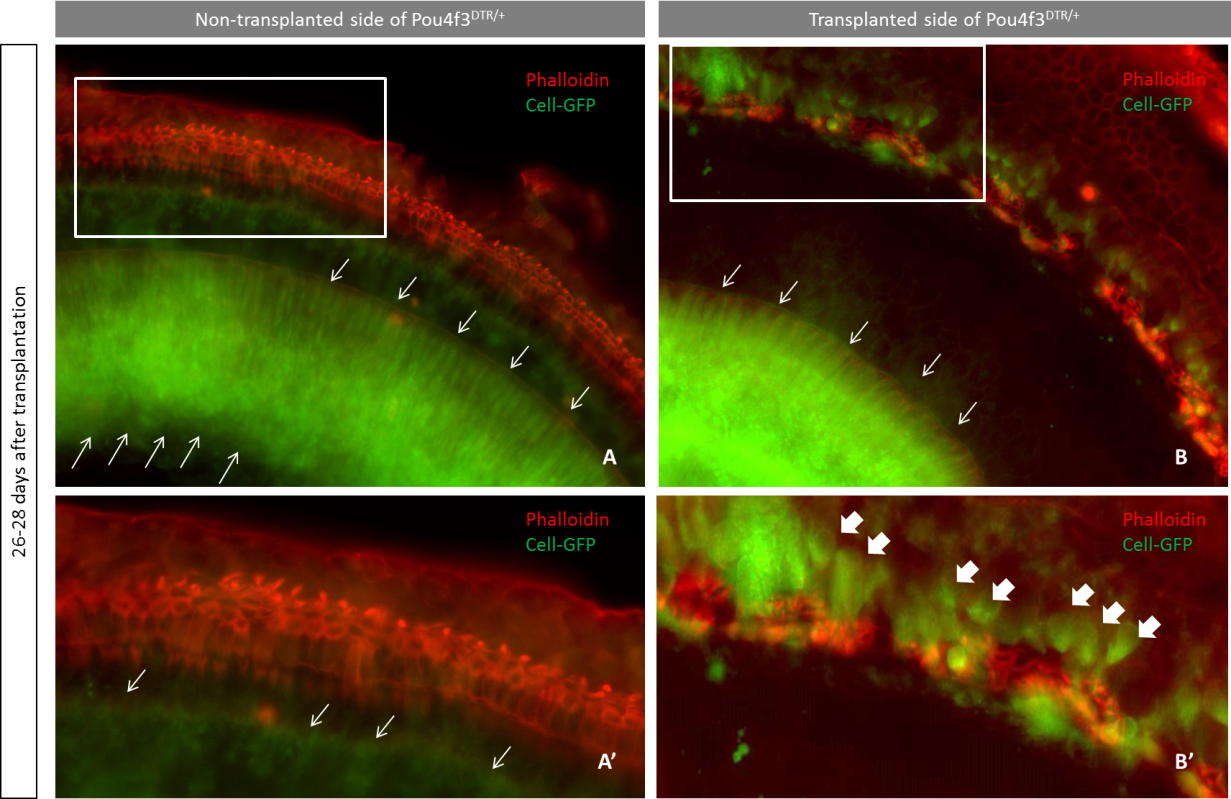


Additional figure 7. Images of the organ of Corti at day 26-28 after cell transplantation in transgenic mice. A; non-transplanted side of the transgenic mouse cochlea, B; transplanted side of the transgenic mouse cochlea. A’ and B’ shows large images of square part in each image. Green indicates autofluorescence (thin arrow) and engrafted human cells (thick arrow). Red indicates phalloining staining. Bars represent 50 μm.


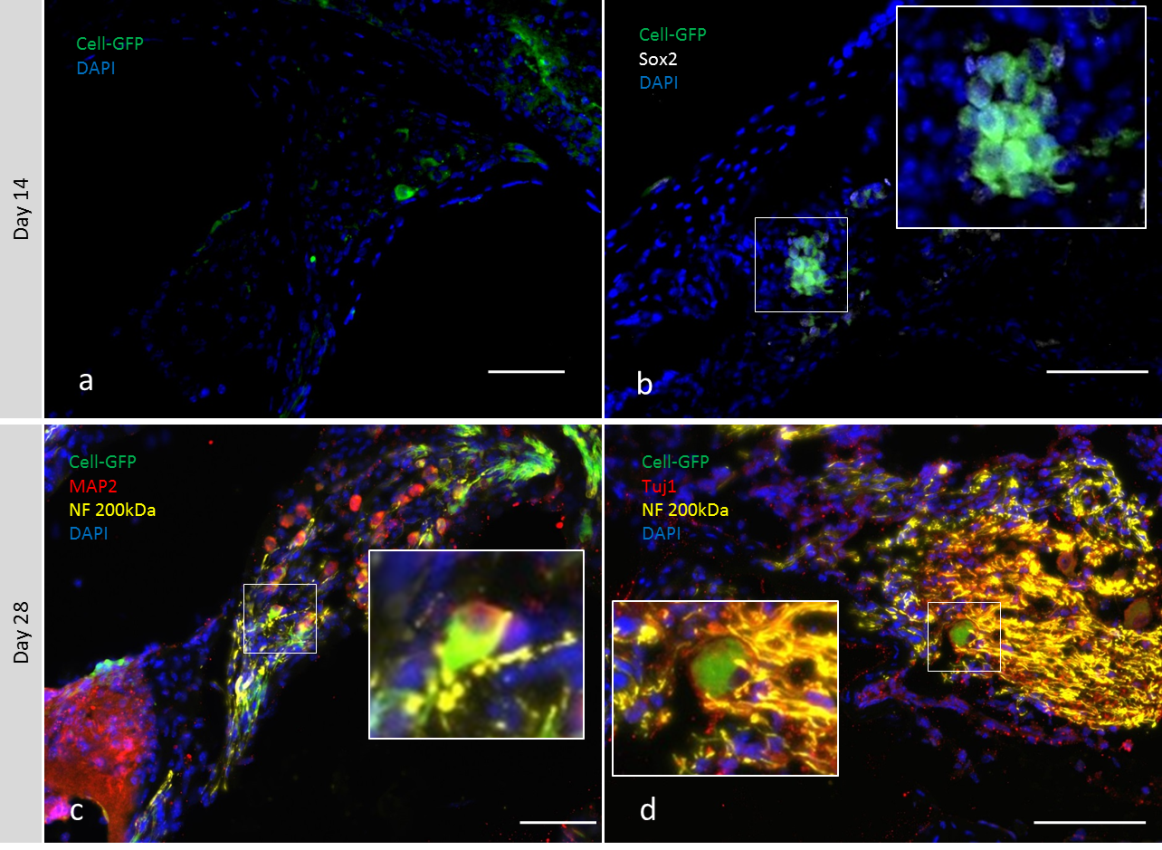


Additional figure 8. Images of the spiral ganglion at day 14 and day 28 after cell transplantation in transgenic mice. Green indicates engrafted human cells in the mouse spiral ganglion. On day 14, some engrafted cells make cluster and those are Sox2 positive (a, b). At day 28, a very few cells are still engrafted in the spiral ganglion (c) and modiolus (d). Green; naïve GFP signal, white; Sox2, red; MAP2 or Tuj1, yellow; NF-200 and Blue; DAPI staining. Bars represent 50 μm.

| **Antibody** | **Species** | **Dilution** | **Manufacturer** | **Cat. No.** |
| --- | --- | --- | --- | --- |
| Pax2 | Mouse monoclonal | 1:1000 | Novus Biologicals | H00005076-M01 |
| Six1 | Rabbit polyclonal | 1:200 | Novus Biologicals | NBP1-84264 |
| Eya1 | Rabbit polyclonal | 1:50 | Proteintech | 22658-1-AP |
| Pax6 | Rabbit polyclonal | 1:50 | BioLegend | 901301 |
| Pax8 | Rabbit polyclonal  Rabbit polyclonal | 1:300  1:100 | Abcam  Proteintech | Ab97477  10336-1-AP |
| Dlx5 | Rabbit monoclonal  Mouse monoclonal | 1:100  1:50 | Abcam  Santa Cruz | Ab109737  sc-398150 |
| Sox2 | Mouse monoclonal | 1:50 | R&D Systems | MAB2018 |
| Oct4 | Mouse monoclonal | 1:50 | Santa Cruz | sc-5279 |

| **Antibody** | **Species** | **Dilution** | **Manufacturer** | **Cat. No.** |
| --- | --- | --- | --- | --- |
| Myo VIIA | Rabbit polyclonal | 1:100-200 | Proteus Bioscience | 25-6790 |
| Sox 2 - AF 647 | Mouse monoclonal | 1:200 | Santa Cruz | sc-365823 AF647 |
| NF 200kDa - AF 555 | Mouse monoclonal | 1:500 | Millipore Sigma | MAB5256A5 |

Additional table 1. Details on antibodies

| **Gene** | **Forward primer** | **Reverse primer** |
| --- | --- | --- |
| Oct4 | 5’- CGA GCA ATT TGC CAA GCT CCT GAA -3’ | 5’- TTC GGG CAC TGC AGG AAC AAA TTC -3’ |
| Six1 | 5’- ACC GCG GCA ACT TCC GTG AG -3’ | 5’- GTG GGT CTG CAG GCC GTG AC -3’ |
| Eya1 | 5’- GCG CGT ACC CAT CCA GGA GC -3’ | 5’- AGA CGG CTA TGC GGG CTG GT -3’ |
| Pax6 | 5’- CGG AGT GAA TCA GTC CGG TG -3’ | 5’- CCG CTT ATA CTG GGC TAT TTT GC -3’ |
| Dlx5 | 5’- CCA GCA TCC GAT CCG GCG AC -3’ | 5’- GTG GGC ATG AGG GTG GTG GC -3’ |
| Pax2 | 5’- TTC CCA GAG TGG TGT GGA CAG TTT -3’ | 5’- ACA ACT GGG TAT GTC TGT GTG CCT -3’ |
| Pax8 | 5’- ACC CCC AAG GTG GTG GAG AAG A -3’  5’- AGC AGG AAA CCC CCG AGG TG -3’ | 5’- CTC GAG GTG GTG CTG GCT GAA G -3’  5’- TTC CTG CCA CCA TGC CTG CG -3’ |
| GAPDH | 5’- ACC ACA GTC CAT GCC ATC AC -3’ | 5’- TCC ACC ACC CTG TTG CTG TA -3’ |

Additional table 2. Primer sequences utilized
